# Supporting autistic doctors in primary care: challenging the myths and misconceptions

**DOI:** 10.3399/bjgp21X716165

**Published:** 2021-06-25

**Authors:** Mary Doherty, Mona Johnson, Carole Buckley

**Affiliations:** Autistic Doctors International; Consultant Anaesthetist, Our Lady’s Hospital, Navan, Meath, Ireland.; Autistic Doctors International; Salaried GP, High Peak, North Derbyshire, UK.; Retired GP and Royal College of General Practitioners Autism Representative.

## AUTISM AS A CLINICAL PRIORITY

From 2014 to 2017 autism was championed by the Royal College of General Practitioners (RCGP). A suite of resources were developed to support GPs in their care of autistic patients.^1^ Engagement on this topic allowed the RCGP and GPs to be represented in national policy and guideline development.

The RCGP continues to promote proper care of autistic patients, their families, and carers. In its position statement on the subject in June 2016^2^ it recognised the essential role general practice plays in caring for this community, their families, and carers. It committed to promoting evidence-based training on autism, and to sign-posting resources that enable equitable access by this group to primary health care.

The Core Capabilities Framework for Supporting Autistic People^3^ and Reasonable Adjustments ‘flag’ on patient records mark out some of the significant progress that has been made. Much remains to be done, and although sensitivities to the needs of autistic patients have improved, the experience and needs of autistic doctors have gone, largely, unrecognised.

## AUTISM PREVALENCE IN HEALTHCARE PRACTITIONERS

Recent prevalence data indicates 1.1% of the UK population is autistic,^4^ although this is widely held to be an underestimate. While we do not yet have prevalence rates for autistic doctors, 1% of GP responders surveyed on this topic by the RCGP clinical priority group^5^ indicated that they were themselves autistic, so informing their care of autistic patients.

Public awareness campaigns,^6^ medical literature,^7^ or the diagnosis of a family member are prompting a ‘lost generation’ of autistic healthcare professionals to recognise their autistic traits,^8^ but it is likely that the majority of autistic doctors remain undiagnosed. Those who have been diagnosed, or who self-identify as autistic, face significant challenges around disclosure. Fearing discrimination, many remain undercover. But now they are finding each other, and this growing community of autistic doctors challenge the assumption that being autistic is incompatible with a medical career.

## AUTISTIC DOCTORS INTERNATIONAL

Autistic Doctors International (ADI) is a peer-support and advocacy group for medical doctors who identify as autistic, or on the autistic spectrum. It was founded in April 2019 with seven members and, to date, has over 300 members, with an associated group for autistic medical students. Over half the members are in the UK, with others from the US, Canada, Australia, and Europe.^9^

GPs make up about a third of ADI’s membership. The second most common specialty for ADI members is psychiatry.^9^ The distribution of members in these highly relational specialties challenges what appear to be common assumptions that — if indeed there are autistic doctors — they are likely to only be found in, so-called, ‘non-patient care’ specialties. In fact, ADI members from specialties such as pathology or radiology are very much in the minority.^9^ Even considering the greater autism awareness among GPs and psychiatrists, the nature of their work challenges the stereotype that autistic people lack empathy.^10^

We find that discussing the existence of autistic doctors elicits one of two contrasting reactions among medical colleagues; disbelief, or a recognition that of course many of our colleagues are autistic, but it’s not something that is openly talked about.

## WORKPLACE CHALLENGES

As the membership grows, narrative themes that were common in the early days — of struggle or difficulty — are shifting. Most members are practising successfully. Now, only a minority fit the category of ‘doctors in difficulty’, though many have, at some point, experienced some sort of difficulty at work.^9^

Difficulties faced by autistic doctors, in our experience, rarely relate to patient care. Indeed, patient contact is usually task-focused or structured around a well-defined role. Rather, trouble arises in the unstructured, unpredictable, or in some other way confusing interactions with colleagues or organisational hierarchy.^11^ Here, social conventions and nuances of neurotypical norms may seem opaque, illogical, or otherwise bewildering. This may mean that interactions with management, training, or regulatory bodies become fraught with misunderstanding, though the issue is entirely understandable when viewed through an autistic lens.

## COMMUNITY, PEER SUPPORT, AND ADVOCACY

In addition to problems arising from communication differences between autistic doctors and colleagues, a preference for predictability and routine, sensory issues, and lack of access to supportive and understanding colleagues appear to be at the root of many of the problems faced by autistic GPs at work — mirroring the needs of autistic anaesthetists.^12^

Given the minority status of this group, many autistic doctors miss out on the understanding or support of colleagues at work or through training. This leads to discrimination, as is illustrated in the Annual Review of Competence Progression (ARCP) outcome excerpt below, received by an ADI member:

*‘The panel regrets to learn of your recent diagnosis of ASD* [autism spectrum disorder]*, but since this is a life-long developmental syndrome which causes permanent impairment of many of the competences required for independent practise as a GP, the panel cannot see how any workplace adaptations could now be put in place to successfully alter your outcome.’*

In this case, the decision to release from training was overturned with the support of ADI. The autistic trainee was reinstated and received an apology.

## FUTURE DIRECTIONS

Stigma and unconscious bias contribute to poor mental health outcomes for autistic people. Camouflaging, or hiding the fact that one is autistic, is associated with increased anxiety, depression,^13^ and suicidality.^14^ Since the rates of mental illness and suicide are considerably higher among doctors than other professions, raising awareness of this hidden minority of autistic doctors is critical.

Although initially established as a place to find belonging, peer support, and community, this vibrant group has expanded its activities. Alongside awareness-raising and advocacy, members are forming collaborations to perform research and gather evidence on autism in doctors, and in the experience of health, illness, and health care for the wider autistic community.

## IMPLICATIONS FOR AUTISTIC PATIENTS

Autistic patients commonly report difficulties communicating with their healthcare providers.^15^ Anecdotally, communication is enhanced between autistic doctors and autistic patients. This is supported through findings that autistic people generally communicate better with other autistic people than with non-autistic people.^16^ It is known that autistic patients face significant health disparities and autistic doctors have a unique perspective on the contributory factors and possible solutions.^17^^,^^18^

Autistic GPs and scholars contributed to the March 2021 issue of the *Australian Journal of General Practitioners*, which focused on neurodiversity.^17^ Their open representation in this publication highlights the heterogeneity of autism and directly challenges the tragedy narrative of autism pervasive in health care.

Changing the culture within medicine so practitioners may disclose without fear of discrimination clearly has positive implications for autistic patients. It is therefore vital the move towards a strengths-based approach to autism is accelerated; the way that autism is framed has a profound impact on outcomes.

## CONCLUSION

As the General Medical Council clearly states: *‘we firmly believe disabled people should be welcomed to the profession and valued for their contribution to patient care’*. It recognises that *‘a diverse population is better served by a diverse workforce that has had similar experiences and understands their needs’*.^19^ Although we recognise the value of diversity within medicine, it is usually considered in terms of race, gender, and sexuality. It is time to openly welcome autism and other aspects of neurodiversity to the conversation, not only to meet statutory duties on equality, or objectives on diversity and inclusion, but because medicine needs all kinds of minds.
